# A comprehensive tool for tumor precision medicine with pharmaco-omics data analysis

**DOI:** 10.3389/fphar.2023.1085765

**Published:** 2023-01-12

**Authors:** Yijun Liu, Fuhu Song, Zhi Li, Liang Chen, Ying Xu, Huiyan Sun, Yi Chang

**Affiliations:** ^1^ School of Artificial Intelligence, Jilin University, Changchun, China; ^2^ Medical Oncology Department, The First Affiliated Hospital of China Medical University, Shenyang, China; ^3^ Department of Computer Science, College of Engineering, Shantou University, Shantou, China; ^4^ Key Laboratory of Intelligent Manufacturing Technology of Ministry of Education, Shantou University, Shantou, China; ^5^ Computational Systems Biology Lab, Department of Biochemistry and Molecular Biology, Institute of Bioinformatics, The University of Georgia, Athens, GA, United States; ^6^ International Center of Future Science, Jilin University, Changchun, China

**Keywords:** cancer precision medicine, drug efficacy, adverse effect, mapping from cell to tissue, web server

## Abstract

**Background:** Cancer precision medicine is an effective strategy to fight cancers by bridging genomics and drug discovery to provide specific treatment for patients with different genetic characteristics. Although some public databases and modelling frameworks have been developed through studies on drug response, most of them only considered the ramifications of the drug on the cell line and the effects on the patient still require a huge amount of work to integrate data from various databases and calculations, especially concerning precision treatment. Furthermore, not only efficacy but also the adverse effects of drugs on patients should be taken into account during cancer treatment. However, the adverse effects as essential indicators of drug safety assessment are always neglected.

**Method:** A holistic estimation explores various drugs’ efficacy levels by calculating their potency both in reversing and enhancing cancer-associated gene expression change. And a method for bridging the gap between cell culture and living tissue estimates the effectiveness of a drug on individual patients through the mappings of various cell lines to each person according to their genetic mutation similarities.

**Result:** We predicted the efficacy of FDA-recommended drugs, taking into account both efficacy and toxicity, and obtained consistent results. We also provided an intuitive and easy-to-use web server called DBPOM (http://www.dbpom.net/, a comprehensive database of pharmaco-omics for cancer precision medicine), which not only integrates the above methods but also provides calculation results on more than 10,000 small molecule compounds and drugs. As a one-stop web server, clinicians and drug researchers can also analyze the overall effect of a drug or a drug combination on cancer patients as well as the biological functions that they target. DBPOM is now public, free to use with no login requirement, and contains all the data and code.

**Conclusion:** Both the positive and negative effects of drugs during precision treatment are essential for practical application of drugs. DBPOM based on the two effects will become a vital resource and analysis platform for drug development, drug mechanism studies and the discovery of new therapies.

## 1 Introduction

Patients of the same cancer type, or even those at the same stage, may respond differently to the same drug (Ginsburg and Phillips, 2018); hence, a comprehensive study of drug screen and the mechanism of drug action on patients with specific pathological and/or physiological conditions will be needed to facilitate improved individualized therapy (Dietel et al., 2013). Pharmaco-omics uses genomics and transcriptomics-based information to help guide individualized drug therapy to maximize drug efficacy and minimize adverse drug reactions (Weinshilboum and Wang, 2017). In the early stages of a drug’s development, its efficacy, toxicity, and sensitivity are typically tested on cell lines (Monks et al., 1991). Currently, the potential treatment outcomes of a drug on a cancer cell line might be assessed by using certain indices like half-maximal inhibitory concentration (IC50). In addition, another increasingly accepted measurement is to compare changes in gene expression signatures of disease samples before and after drug treatment followed by identifying drugs with reversal relationships on disease-associated genes. These studies also demonstrated that the reversal potency of drugs is positively related to the efficacy and therapeutic effect of drugs (Chen et al., 2017). However, the adverse effects of drugs are usually ignored, which is a major concern for both public health and the development of medicines as failing to identify these negative outcomes could lead to significant amounts of morbidity (Jing et al., 2020). Consequently, fully considering both reversed expressions and adverse effects is a highly desirable and meaningful strategy (Nguyen et al., 2021; Vilar and Hripcsak, 2017). What is more, as cancer cells may develop drug resistance, it has been suggested that treatments which employ drug combinations potentially enhance efficacy and reduce toxicity (Wu et al., 2020), and tools providing both toxicity and efficacy analysis of drug combinations can increase the likelihood of clinical success (Ianevski et al., 2020b; Ianevski et al., 2020a). Therefore, the mechanism of synergy and new combination recommendations have acted as a catalyst for intensive studies by academic researchers and pharmaceutical enterprises.

Recently, with the development of high-throughput technology, large amounts of cancer multi-omics data and related therapy data have been released to facilitate cancer-related studies. The Cancer Genome Atlas (TCGA, https://www.cancer.gov/tcga) (Weinstein et al., 2013), molecularly characterized over 20,000 primary cancer and matched normal samples spanning 33 cancer types containing detailed clinical information. The Gene Expression Omnibus database (GEO, https://www.ncbi.nlm.nih.gov/geo/) (Edgar et al., 2002) is a widely used public data repository of the array and sequence-based multi-omics profiles and it stored data not limited to human cancer cells and sample data but multi-species data. The Cancer Cell Line Encyclopedia (CCLE, https://portals.broadinstitute.org/ccle) (Barretina et al., 2012) mainly collected and collated genomics, epigenomics, and transcriptomics data of more than 1,000 cancer cell lines.

Some public databases and modelling frameworks have also been developed and widely used for studies of cancer precision medicine and drug response, such as CMap (Subramanian et al., 2017), GDSC (Yang et al., 2012b), DSigDB (Yoo et al., 2015), SynergyFinder (Ianevski et al., 2020a), and CiDD (San Lucas et al., 2014). While useful, most of these databases and computational tools only consider the effect of the drug on the cell line but not on cancer tissues, resulting in a huge amount of work to integrate data from various databases, and then the calculation is still required to evaluate both the therapeutic and adverse effects on patients, particularly during precision treatment, which makes it extremely difficult for clinicians and drug researchers to utilize these resources (Wakaskar, 2017). Here, we propose the creation of an open-source, comprehensive database of pharmaco-omics for cancer precision medicine that is supported by the collection and storage of drug information, and analysis of the outcomes of drug integrative effect at both a cellular and individual patient level.

In this study, we analysed over 19,000 compound gene expression profiles from CMap database and over 3,000 transcriptomics profiles of tumour and normal samples from TCGA database. To evaluate the effect of a drug on individual patients and not just at a cellular level, we quantified the Reversed and Adverse Drug Effect (RADE) by calculating differential expressions of all the genes between the cancer sample and the normal sample, the cancer cell line with and without drug treatment, respectively, as well as the mapping between cell lines and cancer tissues based on similarities in genetic mutations. By applying RADE to FDA-approved medicines, we found that the potency of most FDA-approved drugs and the ones in clinical trials have positive correlations with RADE recommendations, which suggested the feasibility and potential of RADE as an effective method for identifying drugs with effectiveness and safety. Furthermore, we also analyzed the biological function of reversed and adverse genes when different drug or drug combination treatments are being administered, which can provide possible insights into studies of a drug’s mechanism and play a guiding role in drug development and recommendations. To make our approach more intuitive and easier to use, we developed an easy-to-use web server named DBPOM to store all the calculations of small molecule compounds and drugs across five cancer types based on 28 cell lines for clinicians and drug researchers to search and analyze both the reversed and adverse effects of a drug/drug combination on cancer patients in accordance with the demand for personalized drug analysis.

## 2 Methods

**FIGURE 1:**
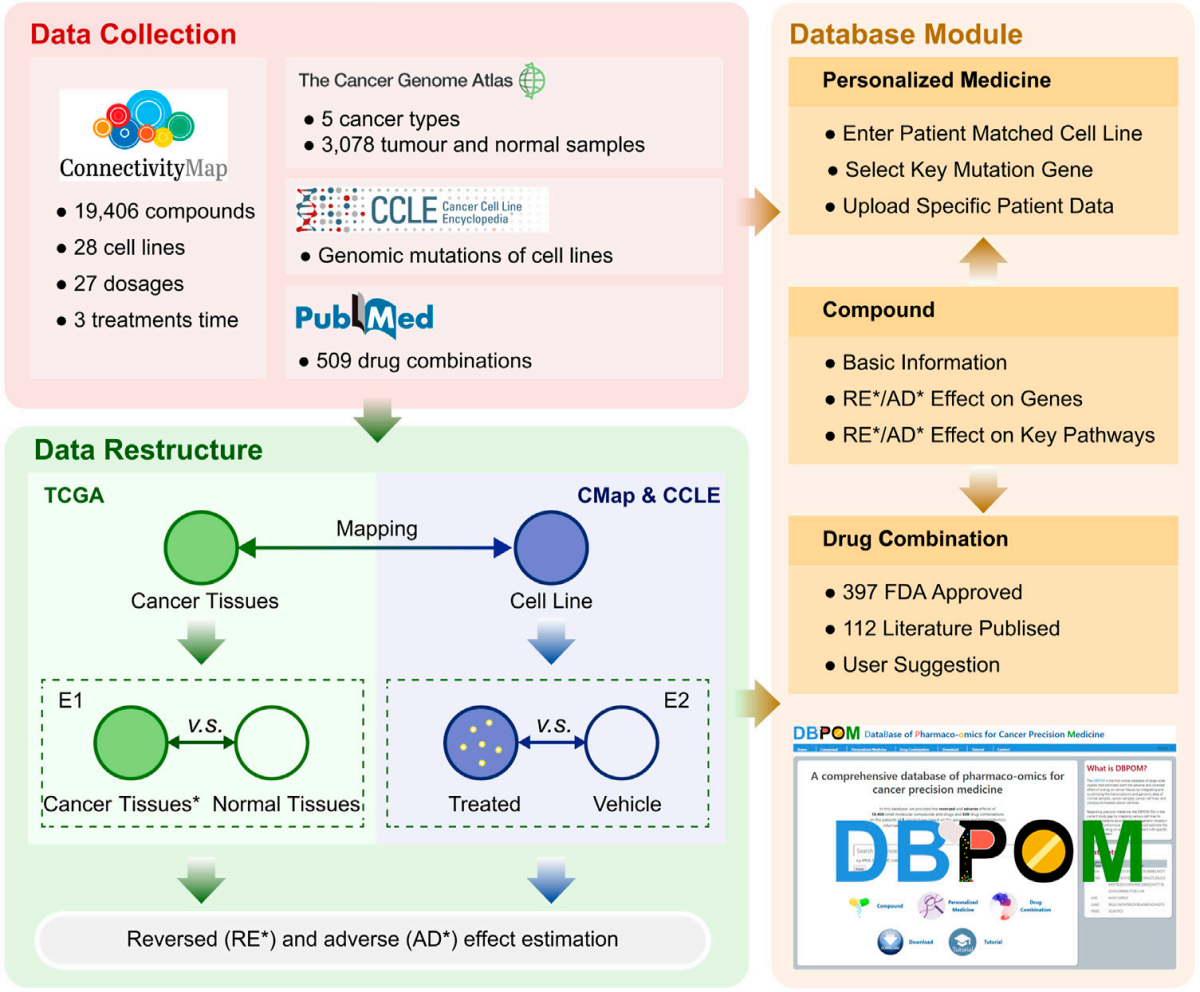
The overall framework includes the methods and DBPOM.

The framework outlined in Figure 1 includes the methods we proposed and functions provided by the web server.

### 2.1 Data source and pre-processing

We collected genomics and transcriptomics data of over 3,000 cancer and normal samples across five cancer types from TCGA, genomics data of 28 relative cell lines from CCLE, gene expression profiles under more than 19,000 compound perturbations from CMap, and over 500 drug combination information from the literature (Cheng et al., 2019). Low-count/low-expressed genes (Raw counts less than 10 in the cancer sample group or normal sample group) were removed from the TCGA dataset for each cancer. Small molecule compounds and drugs from CMap were determined *via* the selected 28 cell lines and the perturbagen treatment included 27 dosages and three treatment durations. After removing 18 pseudogenes, we retained 12,310 expressed genes and their expression under various conditions from CMap. All data was collected before September 2019 and the detailed information of the data resources we used in this paper and calculations can be obtained free of charge in the download centre of DBPOM without login requirement, which was also summarized in Table 1.

**TABLE 1:** Statistical information about the DBPOM.

Key information
19,406 Small molecular compounds and drugs
28 Cell lines
5 Cancer types
27 Dosages
3,078 TCGA cancer samples and normal samples
2,598 Specific mutations with high mutation frequency
397 FDA approved cancer drug combination pairs
112 Literature published cancer drug combination pairs
Total 376,486 files

### 2.2 Differential gene expression analysis and comparisons

Differential gene expression analyses were performed on both cancer tissue samples versus normal tissue samples (E1) from TCGA, and molecular-compound-treated cell lines versus dimethyl sulfoxide (DMSO)-treated cell lines (E2) from the CMap database. In E1, to identify genes associated with carcinogenesis at the gene expression level, DESeq2 package (Love et al., 2014) was used for differential expression analyses between cancer and normal samples in TCGA. We set the threshold as *p*-value = 0.001 and fold-change = 2. For each cancer type, we defined the gene as upregulated (downregulated) and labelled it as 1 (−1) if its fold-change is above two or less than −2 and the *p*-value is less than 0.001; otherwise, it was labelled as 0.

In E2, Eq. 1 was used to estimate fold-change for differential expression analysis between compound-treated and DMSO-treated cell lines in the CMap to evaluate the transcriptomic changes after compound treatment in the cell line. For compound *c*:

$$FC\left(c^{j}\right)=\frac{T\left(c^{j}\right)-D\left(c^{j}\right)}{Max\left\{|D\left(c^{j}\right)|,0.1\right\}}$$ (1)

where *T*(*c* ^*j*^) and *D*(*c* ^*j*^) represent the mRNA expression value of the j-th gene in the compound-treated group and the DMSO-treated group, respectively. *FC*(*c* ^*j*^) represents the fold-change value. In order to prevent overflow, we set 0.1 as the lowest expression value. For each cell line, we defined the gene as upregulated (downregulated) and labelled it 1 (−1) if its fold-change is above 2 or less than −2 when comparing compounds with DMSO-treated cell lines; otherwise, it was labelled as 0.

### 2.3 Disease gene expression signatures and assessment

We proposed a definition for distinguishing the reversed and adverse genes on cancer samples after a specified compound or drug treatment by comparing the data stemming from the two groups of differential gene expression results (E1 and E2), as shown in Figure 1. Both effectiveness and side effects are all considered in this definition, by comparing the transcriptomic changes of genes expressed in cancer tissues compared to normal tissues, and genes significantly altered in cell lines after drug treatment. When a cell line is treated with a compound, if the effect on a gene is reversed, meaning it is upregulated in E1 and downregulated in E2 or *vice versa*, we consider it to have a reversed effect on the mentioned gene, which means that the treatment has the potential to correct the gene-related biological process (van Noort et al., 2014; Chen et al., 2017). Otherwise, if a gene is marked as 1 (E1) ∼1(E2), −1(E1) ∼-1(E2), 0 (E1) ∼1 (E2) or 0 (E1) ∼-1 (E2), with 0 standing for no change, the compound is considered to have an adverse effect on this specific gene, which suggests that the treatment has the potential to promote deterioration of the gene-related biological process (Heinloth et al., 2004). For each compound, if it reverses the expression of a cancer-associated gene, it is thought to be effective on the gene. On the contrary, if the gene expression substantially deviates after treatment with the compound, we believe that the drug has an adverse effect on the gene. It should be noted that the adverse effects defined here are different from the pharmacological adverse reactions in clinical trials and we set different thresholds for reversed and reverse effects based on the analysis of the distribution of FC values for reversed and adverse genes (Supplementary Figure S1). The formulaic expression is defined as:

$$\begin{array}{c} \text{ Reversed effect }\left\{\begin{array}{c} InE1:FC\left(c^{j}\right)>2\text{ and }InE2:FC\left(c^{j}\right)<-2\;\\ InE1:FC\left(c^{j}\right)<-2\text{ and }InE2:FC\left(c^{j}\right)>2\; \end{array}\right.\\ \text{ Adverse effect }\left\{\begin{array}{c} InE1:FC\left(c^{j}\right)=0\text{ and }InE2:\left|FC\left(c^{j}\right)\right|>15\;\\ InE1:FC\left(c^{j}\right)>2\text{ and }InE2:FC\left(c^{j}\right)>15\;\\ InE1:FC\left(c^{j}\right)<-2\text{ and }InE2:FC\left(c^{j}\right)<-15\; \end{array}\right. \end{array}$$ (2)

To estimate the overall effect of a drug on each patient, we quantified both the reversed and adverse effects of all the genes as RADE (the reversed and adverse drug effect) to comprehensively assess both the effectiveness and safety of each compound. RADE considers the number of reversed and adverse genes of tissues after compound treatment, specifically, ${\text{RADE}}_{c}^{k}$ denotes the score of compound *c* treating on cell line *k*:

$${\text{RADE}}_{c}^{k}=\frac{\#Ad^{k}\left(c\right)}{\#Re^{k}\left(c\right)}*\frac{\#Mu\left(k\right)}{\#Re^{k}\left(c\right)}$$ (3)

where sign *#* represents the number of elements in the set, *Ad* ^*k*^(*c*) and Re^*k*^(*c*) are the set of adverse and reversed genes in cell line *k* under the compound *c* treatment, respectively. *Mu*(*k*) is the set of genes whose mRNA expression significantly changed in cancer to which cell line *k* belongs, i.e., the absolute fold-change above two and *p*-value less than 0.001 in E1. In Eq. 3, the first term is the ratio of deteriorating and corrected transcriptomic changes, which illustrates the power of the drug, and the second is the ratio of significant transcriptomic changes of cancer and the corrected transcriptomic changes after the drug treatment, which underlines the degree of reversal effect of compound *c* on this cancer. The lower RADE indicates the higher effectiveness and safety of the compound on the cell line.

### 2.4 Mapping individual mutation phenotype to cancer cell line

Genetic mutations of different cancer cell lines of the same cancer type vary, which results in them responding divergently to the same drug (Zhao et al., 2020). Meanwhile, as an important reference for molecular subtypes, the genomic mutation could reflect the individual characteristics of different patients with the same type of cancer. Therefore, it is helpful for precision medicine that starts with the estimation of the similarity between cancer cell lines and genetic mutations of patients followed by personalised treatment recommendations based on the cell line’s response to drugs.

We collected the genomic mutation information of cell lines and cancer patients from the CCLE and TCGA database, and defined the similarity score of a cancer cell line and a cancer tissue from the same cancer type in the following manner:

$${\text{Match}}_{k}\left(s\right)=\frac{\#\;DE\left(s\right)\cap Mucl\left(k\right)}{\#Mucl\left(k\right)}$$ (4)

$$L\left(s\right)=\underset{k}{\arg\max}{\text{Match}}_{k}\left(s\right)$$ (5)

where sign *#* represents the number of elements in the set, Match_*k*_(*s*) is the matching score of sample *s* on cell line *k*, *DE*(*s*) is the set of mutation genes in sample *s*, and *Mucl*(*k*) is the set of mutation genes of cell line *k*. *L*(*s*) is the cell line with the highest matching score of sample *s*. Sample *c* was roughly labelled as a cancer type in TCGA and is re-labelled as a cell line according to Eqs 4, 5.

Thus, there are three reference options with different sample selection strategies in E1 by the method of estimating the similarity of cancer samples to the mutation phenotype of cell line *k*.

(i) TCGA versus CMap: in E1, all the cancer samples of the selected cancer type in TCGA are utilized. While this is the general strategy in current research, it does not consider individual differences. The web server provides this option in case some users need it.
(ii) subTCGA-mutation versus CMap: in E1, the subset of cancer samples in TCGA which have high matching scores with cell line *k* are utilized. Compared with the last strategy, this one uses the sample matching Eqs 4, 5 and provides a more fine-grained comparison and result. In practice, each cancer patient is first assigned to the most appropriate sample group based on the similarity of the mutation and then the effect of the drug on them is analysed. DBPOM provides users with this option to suit diverse requirements.
(iii) subTCGA-specific versus CMap: E1 utilizes a subset of cancer samples with the specific mutation that the user is highly concerned with from TCGA, which match the specific mutation that the user concerns with of cell line *k* in E2. We proposed this strategy and expected to get a reasonable evaluation of drug effects that could then assist precision medicine.

### 2.5 Pathway enrichment analysis

To better disclose the real effects of drugs on cancer samples, we performed functional analysis on the adverse and reversed genes of each perturbation experiment to find the main biological pathways of KEGG, GO, PID, and REACTOME databases that the compound potentially targets. The statistical significance of the enrichment analysis is obtained based on the hypergeometric test below:

$$P=1-\overset{m-1}{\underset{i=0}{\sum }}\frac{\left(\begin{array}{c} M\\ i \end{array}\right)\left(\begin{array}{c} N-M\\ n-i \end{array}\right)}{\left(\begin{array}{c} N\\ n \end{array}\right)}$$ (6)

Here, *N* is the number of all genes in the transcriptome, *M* is the number of genes in the gene set to be detected, *n* is the number of genes intersecting with the adverse/reversed gene set in *N*, *m* is the number of adverse/reversed genes in the pathway. *P* represents the enrichment significance of adverse/reversed genes on the pathway and genes and *P* of 0.05 is the threshold indicating a statistically significant association. FDR (False Discovery Rate) multiple testing correction (Benjamini and Hochberg, 1995) is used in the hypergeometric test to reduce the number of false-positive conclusions.

## 3 Results

This study aimed at assessing the effects of treatments on cancer patients by integrating the gene signatures of different perturbation experiments on cell lines, particularly cancer types or subtypes associated with genes. To comprehensively represent our results (for almost 20,000 small molecules, 28 cell lines) and provide researchers with a more flexible scenario to utilise our proposed methods, we designed the DBPOM web server with three modules including the compound effect on cancer samples, patient-centric precision medicine, and drug effect comparison of known/unknown drug combination pairs.

### 3.1 Search by compound

**FIGURE 2:**
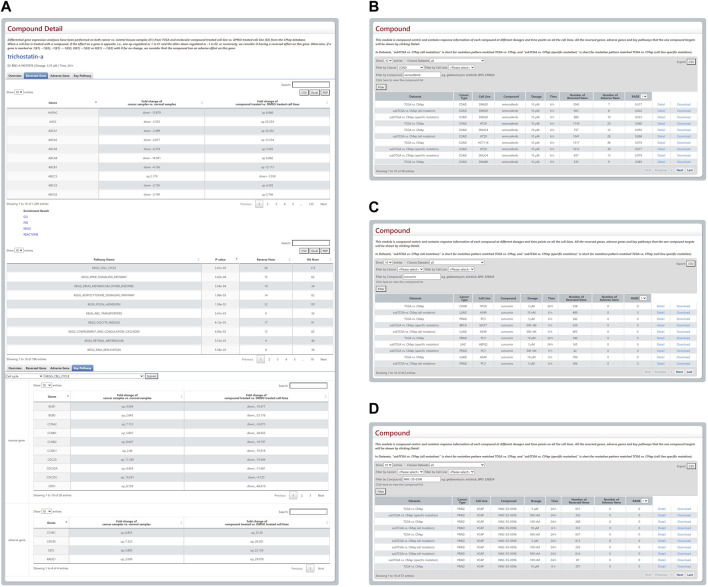
Outline and statistical analysis of Compound module. **(A)** The compound detail page includes concise information, details of reversed and adverse genes, and pathway enrichment analysis. **(B–D)** Three responsive tables of users’ search results have been employed to prove the rationality of the RADE scores raised by the DBPOM.

This module is compound-centric and contains response information of each compound at different dosages and treatment durations when applied to all cell lines. As shown in Figure 2A, when a user selects a compound, the response information has two levels. One is the whole cell level. Users can bring up the detail page for a more in-depth understanding and pathway enrichment analyses, which are provided for analyzing the functions of reversed and adverse genes. The other stratum is the pathway level. The database provides a panel focusing on the treatment effects of drugs or compounds on the genes involved in cell proliferation, cell death and 11 other key pathways of high attention for clinicians and drug researchers, including cell cycle and apoptosis regulation. The module also offers information about the compound like its IC50 and structure from other databases. Users can choose a cell line in isolation to ascertain the responses of all compounds to this cell line at different dosages and treatment durations. According to our calculation formula, a drug with a higher score is thought to have a lower possibility of being both effective and safe on a certain cell line.

We found that several drugs that were FDA-approved, and some that are being employed in clinical trials for cancer treatment, have low RADE values in our database, which demonstrates that the RADB reasonably has the potential to evaluate the drug’s efficiency and safety. For example, vemurafenib is an FDA-approved drug used for the treatment of metastatic melanoma with a mutation on the BRAF in the valine located in the exon 15 at codon 600 (Luke and Hodi, 2012). When combined with standard-of-care or novel-targeted therapies, it is also reported that vemurafenib is effective on colorectal cancer (Yang et al., 2012a). Via DBPOM, we discovered that the RADE value of vemurafenib acting on COAD generally is extremely low as well (Figure 2B). As another example, curcumin is a drug that has been used in clinical trials and been investigated for the treatment and supportive care of clinical conditions, including proteinuria, breast cancer, multiple myeloma, and non-small-cell lung cancer (Gupta et al., 2013). The RADE value of curcumin is also very low in DBPOM (Figure 2C). Moreover, NNC-55-0396 is a molecular compound still in the drug development phase and reportedly could prevent human cancer cell proliferation and induce cancer cell apoptosis as a result of its ability to inhibit the function of T-type Ca^2+^ channels (Huang et al., 2015); the experimental results are also in good agreement with the value we proposed (Figure 2D).

### 3.2 Personalized medicine

**FIGURE 3:**
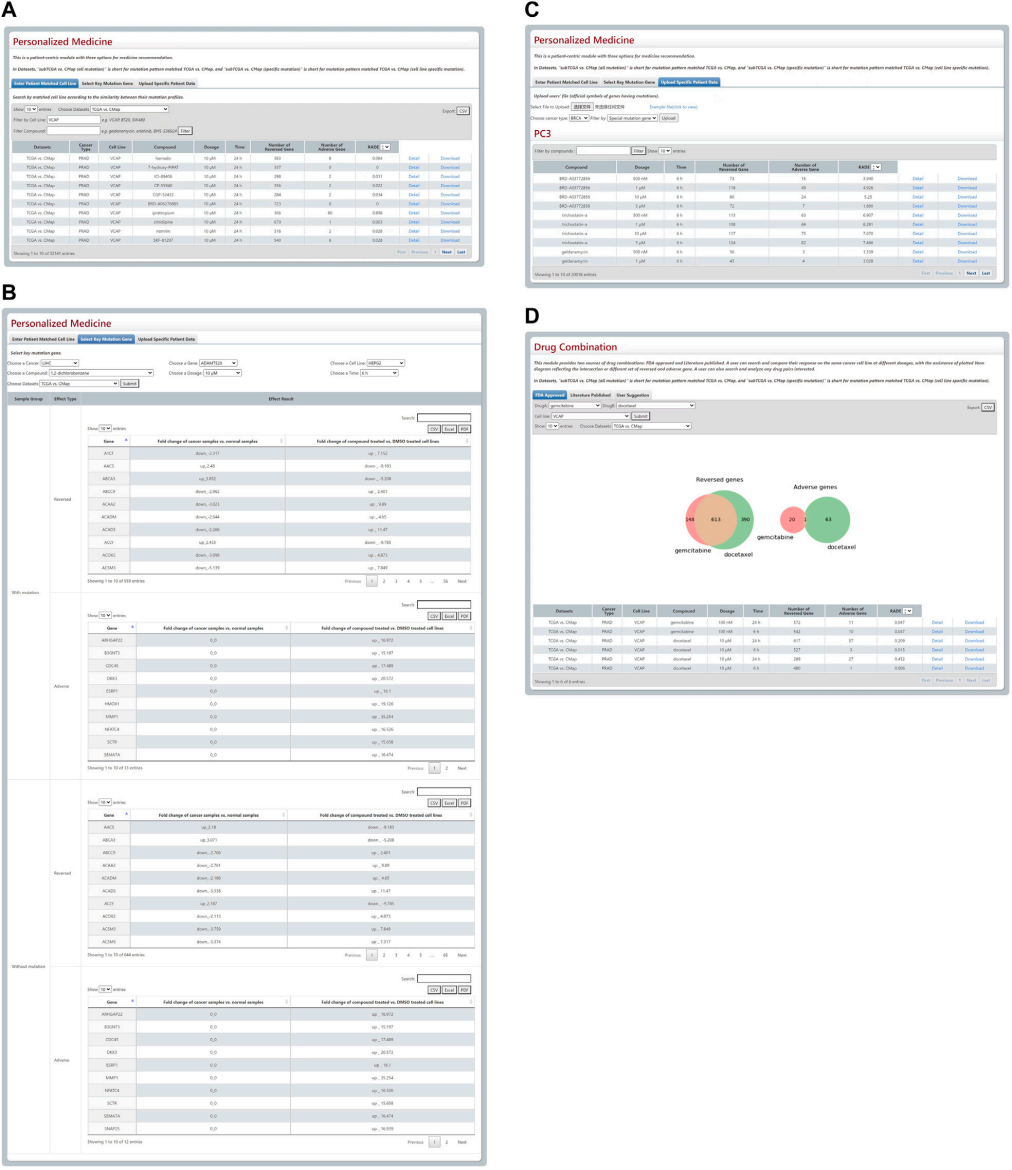
Results from Personalized Medicine and Drug Combination modules. **(A–C)** The three modules for personalized medicine. **(D)** The Venn plot and search results of gemcitabine and docetaxel.

There is a patient-centric module that contains three options for precision medicine in DBPOM. The first is for an optimal match between cell lines and cancer samples according to the similarity score of their overall mutation patterns. When a cell line is chosen, the module matches the referenced cancer samples with the cell line and performs the aforementioned analysis (Figure 3A). The second relates to the specific mutation that a user is interested in. When a certain mutation and a drug are selected, this module compares both the reversed and adverse effects of this drug on cancer patients versus ones without the mutation (Figure 3B). For each cancer, we list the genes with high genetic mutation rates. Finally, the user can also submit his/her own data regarding a patient’s mutation profile for analysis (Figure 3C).

### 3.3 Drug combination

This module provides FDA-approved, text-mining-validated drug pairs. For each pair, the user can search and compare their response to the same cancer cell line in a different physical environment with the assistance of a plotted Venn diagram that reflects the intersection or a different set of reversed and adverse genes. Furthermore, they can also search and investigate any drug pairs they are interested in. For instance, the gemcitabine and docetaxel combination approved by FDA is used for the therapy of breast, ovarian, and non-small-cell lung cancer and these two drugs’ reversed and adverse genes on VCAP cell line are shown in a Venn plot (Figure 3D).

### 3.4 Easy download

This module allows easy access to all the reversed and adverse effect data of 19,406 small molecular compounds and drugs, and 509 drug combinations on the 12,310 genes of cancer patients across five cancer types and 28 cell lines with partial/complete download options. DBPOM displays succinct information about the gene signatures of compounds and drugs and detailed description files can be downloaded in this module.

### 3.5 Database and web interface service and implementation

DBPOM affords researchers an online analysis capability for compounds, and they can easily download their query or calculation results from any module. The data in the DBPOM was all stored and managed by MySQL, and the web interface was implemented using HTML and JavaScript. In addition, all of the data analysis work was completed with R and Python.

## 4 Discussion and future directions

We proposed a series of methods to explore the effects of a potential drug on cancer patients *via* the use of genomics and transcriptomics-based information. Compared with previous prediction methods, we focused on both the reversed and adverse genomic effects of a drug to comprehensively assess the effectiveness and safety of the drug. We also designed an easy-to-use web server called DBPOM which offers users easy access to our results and the use of our methods for diverse requirements. Clinicians and drug researchers could search and analyze both the reversed and adverse effects of a drug or drug combination on cancer patients according to their needs for personalized drug analysis, which is unlike other similar databases or web servers that focus solely on cell lines. In addition, DBPOM provides a worthwhile way to compare the effect of different drugs through the analysis of gene expression change, meaning it could assist in speeding up the process of drug development, facilitate new uses of old drugs, and act as a catalyst for the discovery of new therapies. As far as we know, DBPOM is the first large-scale database to estimate adverse gene expression changes after drug treatment.

Considering the differences in the efficacy and safety requirements of drugs at different stages of cancer, we also performed a systematic analysis of RADB scores for the recommended drug for lung cancer and found consistency between the existing clinical results and RADB. For example, cisplatin (Group, 2004), gefitinib (Giaccone, 2004), and pemetrexed (Rossi et al., 2009) are three FDA-approved drugs for lung cancer treatment and rank high in the database (in detail, cisplatin reverses 676 genes, gefitinib reverses 646 genes, and pemetrexed reverses 588 genes and they all do not have any adverse effect on any genes and rank 1/20931). Thiocolchicoside, which has been shown to increase the risk of developing cancer^1^, has reversed effects on 1,165 genes and adverse effects on 217 genes and ranks 20,928/20,931 according to 2.26 RADB score. In addition, although topotecan is recognised as an effective treatment for lung cancer, it shows a high adverse effect score in our database (in detail, it has reversed effects on 1,165 genes and adverse effects on 217 genes and ranks 18,415/20,931 with 0.4 RADB score), which is in line with the existing drug instruction that recommends it as a treatment for small cell lung cancer that has come back or spread and the effectiveness is more important for such patients^2^. As mentioned above, some drugs do not have any adverse effects on any genes, i.e., *#Ad* ^*k*^(*c*) = 0, and RADBs are set as 0. Therefore, although RADB can assess both the safety and effectiveness of drugs, it also makes sense to take the number of reversed and adverse genes into account.

In the future, We will continue to collect more data relating to other cancer types, cell lines, compounds, and approved drug combinations to enrich DBPOM. We will also develop and import more computational tools for reversed effects, adverse effects and their comprehensive effect estimation as well as extend the capabilities for precision medicine recommendations. We expect the DBPOM to become a vital resource and analysis platform for drug development, drug mechanism studies, and new therapy discoveries.

## Data Availability

The original contributions presented in the study are included in the article/Supplementary Material, further inquiries can be directed to the corresponding authors.

## Footnotes

1
https://www.ema.europa.eu/en/news/european-medicines-agency-recommends-restricting-use-thiocolchicoside-mouth-injection

2
https://www.cancerresearchuk.org/about-cancer/cancer-in-general/treatment/cancer-drugs/drugs/topotecan
