# Supplementary material for: A comprehensive tool for tumor precision medicine with pharmaco-omics data analysis
Source: Front Pharmacol. 2023 Jan 12;14:1085765. doi: 10.3389/fphar.2023.1085765 (PMC9878337; doi:10.3389/fphar.2023.1085765)
Supplement: Supplementary file 1 [file DataSheet1.PDF]

Supplementary Material

1 PARAMETER ANALYSIS OF DISEASE GENE EXPRESSION SIGNATURES AND ASSESSMENT

The differential gene expression analysis on cancer versus control tissue samples from TCGA database labelled as E1, and the threshold setting is the absolute of fold-change (FC) more than 2 and the p-value less than 0.001, which is generally used in bioinformatics studies to identify genes with significant transcriptomic changes.

The differential gene expression analyses on molecular-compound-treated cell lines versus dimethyl sulfoxide (DMSO)-treated cell lines from the CMap database are labelled as E2. The threshold of E2 is set as 2 for reversed genes and set as 15 for adverse genes as illustrated in Equation 2. Different thresholds are determined by analyzing the distribution of FC values of genes and the histogram plots of distribution for 5 cancers are shown in Figure S1. We first removed genes with absolute FC less than 2 (such genes are generally considered as not changed significantly), then randomly selected 1,000,000 fold-change values of genes in E2, and draw the histogram plots of the frequency and density of the values. For each cancer, the plots include distributions of the whole 1,000,000 FCs, the FCs of reversed genes (genes with opposite labels in E1 and E2) in the whole FC values, and the FCs of adverse genes (genes with the same labels in E1 and E2, and genes that have no significant change in E1 but $|FC| > 2$ in E2) in the whole FC values.

From Figure S1, the number of reversed genes is far less than the number of adverse genes, which suggests that it is more difficult for a drug to reverse cancer-associated gene expression changes compared with promoting the changes. Taking PRAD as an example as shown in Figure S1A, which has the least proportion of reversed genes in total FCs compared with the other four cancers, the genes marked as reversed are only about 63,000 (Figure.S1A(b)). By increasing the defined FC threshold for side effect genes to 15, it is possible to equalise the number of adverse genes with the number of reversed genes (Figure.S1A(d)), which also ensures that the adverse effect of selected genes is really a continuous deterioration under the drug treatment. So in E2, we set the threshold as 2 for reversed genes and the threshold as 15 for adverse genes to balance the number of genes with different labels and increase the likelihood of genes labelled as adverse with actual adverse effects.

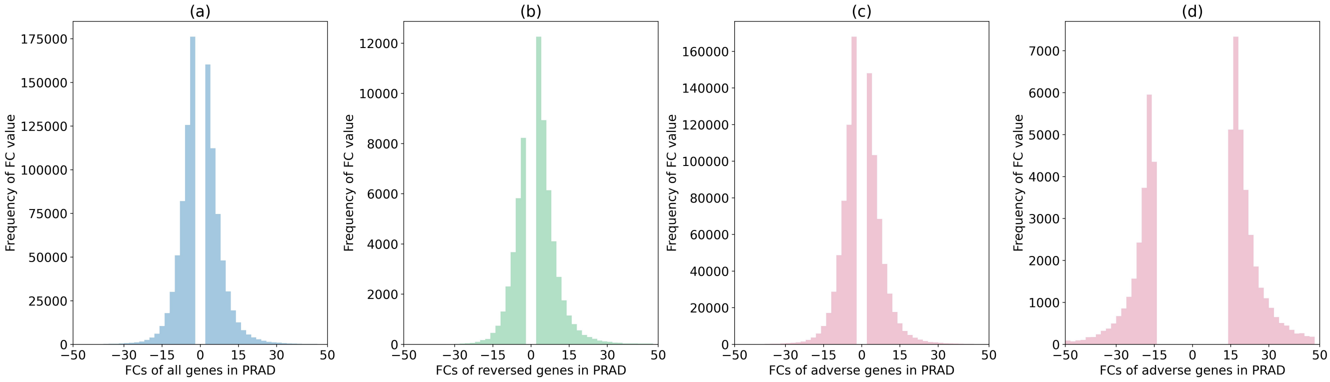

Figure S1A. The distribution of FCs in PRAD. **(a)** The frequency distribution of FCs randomly selected. **(b)** The frequency distribution of FCs of reversed genes in the selected FCs. **(c)&(d)** The frequency distribution of FCs of adverse genes in the selected FCs with the threshold of 2 and 15.

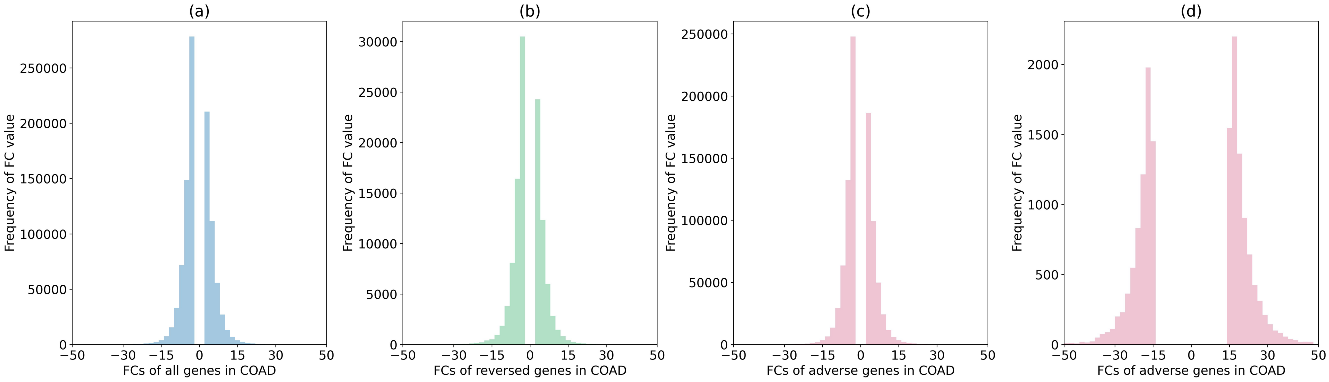

Figure S1B. The distribution of FCs in COAD. **(a)** The frequency distribution of FCs randomly selected. **(b)** The frequency distribution of FCs of reversed genes in the selected FCs. **(c)&(d)** The frequency distribution of FCs of adverse genes in the selected FCs with the threshold of 2 and 15.

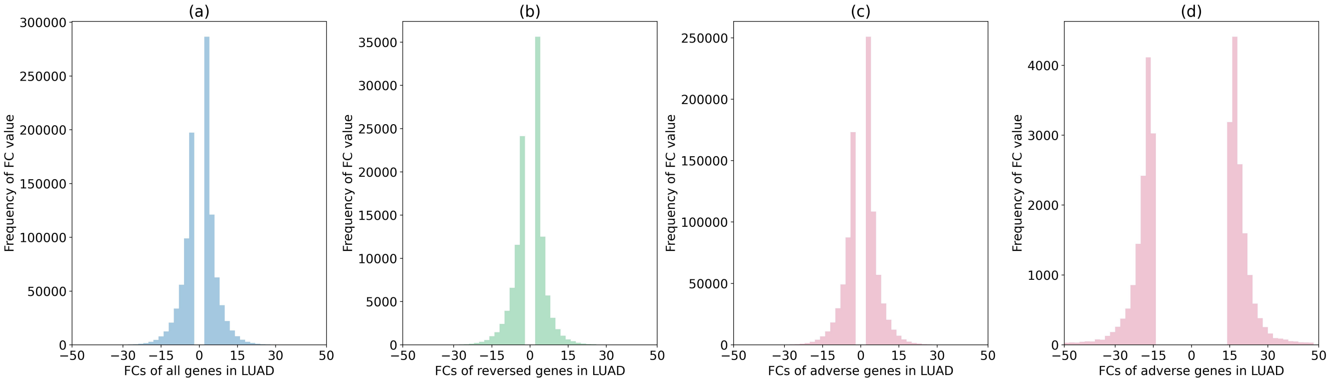

Figure S1C. The distribution of FCs in LUAD. **(a)** The frequency distribution of FCs randomly selected. **(b)** The frequency distribution of FCs of reversed genes in the selected FCs. **(c)&(d)** The frequency distribution of FCs of adverse genes in the selected FCs with the threshold of 2 and 15.

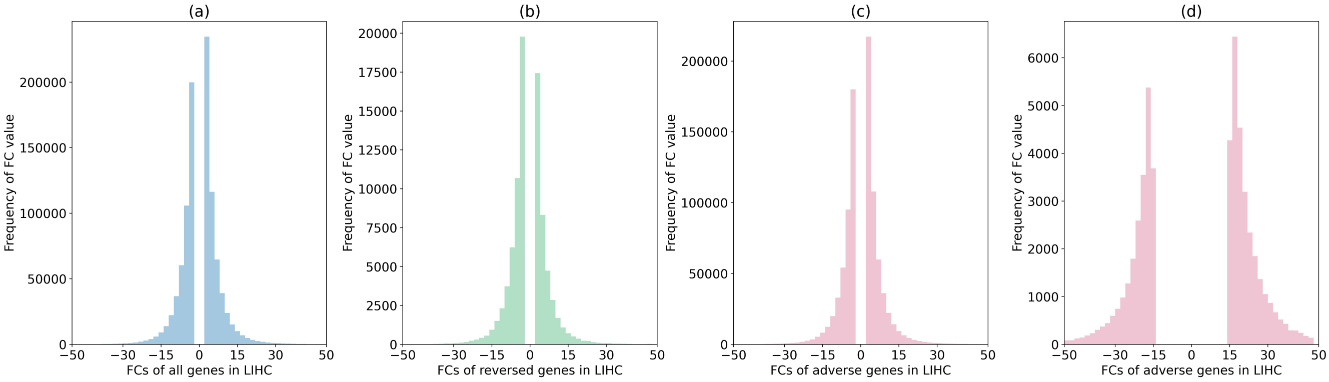

Figure S1D. The distribution of FCs in LIHC. **(a)** The frequency distribution of FCs randomly selected. **(b)** The frequency distribution of FCs of reversed genes in the selected FCs. **(c)&(d)** The frequency distribution of FCs of adverse genes in the selected FCs with the threshold of 2 and 15.

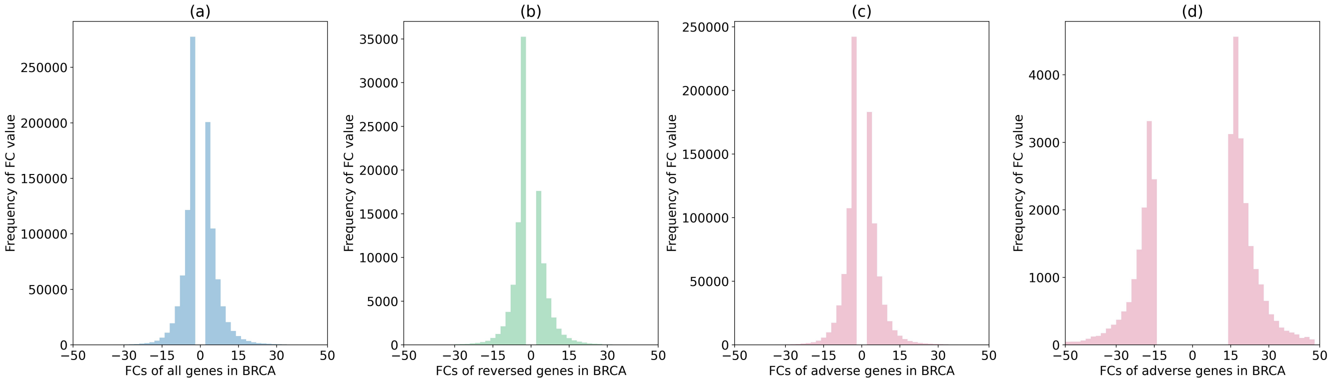

Figure S1E. The distribution of FCs in BRCA. **(a)** The frequency distribution of FCs randomly selected. **(b)** The frequency distribution of FCs of reversed genes in the selected FCs. **(c)&(d)** The frequency distribution of FCs of adverse genes in the selected FCs with the threshold of 2 and 15.

Figure S1: The distribution of FCs in five cancers.
